# Does Feeding Management Make a Difference to Behavioural Activities and Welfare of Horses Reared for Meat Production?

**DOI:** 10.3390/ani12141740

**Published:** 2022-07-06

**Authors:** Federica Raspa, Martina Tarantola, Edlira Muca, Domenico Bergero, Dominga Soglia, Damiano Cavallini, Ingrid Vervuert, Clara Bordin, Pasquale De Palo, Emanuela Valle

**Affiliations:** 1 Department of Veterinary Sciences, University of Turin, Largo Paolo Braccini 2, 10095 Grugliasco, Italy; martina.tarantola@unito.it (M.T.); edlira.muca@unito.it (E.M.); domenico.bergero@unito.it (D.B.); dominga.soglia@unito.it (D.S.); clara.bordin@edu.unito.it (C.B.); emanuela.valle@unito.it (E.V.); 2 Department of Veterinary Sciences, University of Bologna, 40064 Ozzano dell’Emilia, Italy; damiano.cavallini@unibo.it; 3 Faculty of Veterinary Medicine, Leipzig University, 04103 Leipzig, Germany; ingrid.vervuert@vetmed.uni-leipzig.de; 4 Department of Veterinary Medicine, University of Bari, 70010 Valenzano, Italy; pasquale.depalo@uniba.it

**Keywords:** equine, diet, welfare, forage, concentrate

## Abstract

**Simple Summary:**

Identifying effective and economically feasible changes to apply at the farming level to improve animal welfare are of great importance. Horses reared for meat production are conventionally fed high amounts of concentrates rich in starch and simple sugars; however, horses are herbivores and adapted to eat a fibre-based diet. The aim of the present study was to assess the effects of two different feeding management systems on the behavioural activities and subsequent welfare of horses reared for meat purposes. Our findings provide new insights into the positive consequences of feeding horses reared for meat production on a fibre-based diet in terms of both welfare and farming economics. This change in feed management allows horses to express a more natural time budget, spending more time expressing feeding behaviour, which improves horse welfare and reduces energy expenditure in the form of excitable behaviours.

**Abstract:**

Horses reared for meat production are generally fed a diet rich in starch with the aim of maximizing production performances. This study evaluated the effects of two feeding management systems on horse welfare by analysing the relative time spent engaged in different behavioural activities. Nineteen Bardigiano horses aged 14.3 ± 0.7 months were randomly assigned to one of two group pens: one group was fed high amounts of starch-rich concentrates (HCG; *n* = 10), the other was fed a fibre-based diet (HFG; *n* = 9). Behavioural activities performed by each horse were video-recorded over a 96-h period. A scan sampling process (*n* = 144 scans/horse/day; total *n* of scans sampled = 10,368) was used, and the scans were analysed according to a specific ethogram. The mean frequency (%/24 h) spent exhibiting each behavioural activity was calculated to obtain the time budget. After checking for normality (Shapiro–Wilk test), Student’s *t* tests (normally distributed data) and Mann–Whitney tests (not normally distributed data) were used to compare the time budgets of the two groups of horses (HCG vs. HFG). Principal Component Analysis (PCA) was applied to identify the components explaining the variability in behavioural activities between the two groups. K-means cluster analysis subsequently confirmed the PCA results. The behavioural activities associated with feeding horses a fibre-based diet correlated with better horse welfare compared with feeding horses a starch-based diet. Feeding horses a fibre-based diet resulted advantageous from both the welfare and economic perspective; it allowed horses to spend more time expressing feeding behaviours and reduced energy expenditure in the form of excitable, or “fizzy”, behaviours.

## 1. Introduction

Consumer interest in horse meat as an alternative source of animal protein is growing in a number of European countries thanks to its well-recognised nutritional value. Horse meat is characterised by its low intramuscular fat, low cholesterol, and high content of bioavailable iron (3.89 mg per 100 g meat), which is nearly double that of other red meats [1]. Spain is the biggest horse meat producer (17%) within the EU, closely followed by Italy (16%), Romania (14%), Poland (11%) and France (8.2%) [2]. However, despite the fact that more than half a million horses are slaughtered in Europe each year [3], there is a lack of studies directed at assessing and safeguarding the welfare of horses reared for meat production, particularly during their fattening period which is usually carried out in indoor group pens. Instead, the scientific literature has mainly focused on the meat itself; for example, considering its consumption statistics and nutritional properties. In addition, there are no standardised farming conditions for the breeding of these animals [4], despite it being known that horses are often farmed in intensive systems characterised by high stocking densities, group pens, and an intensive feeding management [5]. An intensive feed management strategy involves giving high amounts of starch-rich concentrates (7–8 kg/horse/day) to reduce the length of the fattening period and increase meat production performances [4,5,6]. However, the application of this type of feed management in horses is not based on scientific evidence, but instead seems to be a convention deemed to be appropriate by farmers on the basis that “it has always been done that way”. Yet, it is well known that feeding horses starch-rich diets can negatively affect the welfare of these animals [5] by affecting their gastrointestinal health; for example, by increasing their risk for gastric ulcers and colic [7], and by influencing their behavioural activities [8,9]. This latter aspect is particularly important from a welfare assessment point of view, which, according to the Five Domains Model proposed by Mellor [10], needs to involve the study of the animal behaviour. Indeed, a reduction in a horse’s behavioural repertoire and/or a change in its time budget can reflect poor or inadequate horse welfare [9]. Considering that all domestic breeds of horse continue to express the species-specific behaviours of their wild ancestors [11,12], a behaviour that all horses need to engage in for their welfare is the one that they would naturally dedicate 16–18 h of their day to [13,14]: foraging. However, meeting this need is frequently not considered a priority by horse owners and thus it is generally not met, especially in those that are farmed.

Whereas promoting and improving welfare assessment in horses reared for meat production are both crucial in order to safeguard their welfare [5], it is equally important to propose feasible solutions to farmers which enable them to improve animal welfare without negatively influencing the economics of their businesses [15]. Respecting the horse’s physiological need for foraging could be one economically feasible solution that safeguards horse welfare without reducing productivity. In a recent study, Raspa and colleagues [16] found that feeding horses reared for meat production high amounts of concentrates based on cereal grains did not result in larger bodyweight gains or better meat quality traits compared with horses fed a fibre-based diet. It was also shown that increasing the nutritive level of the diet did not result in any positive effects on meat quality traits or horse carcass weights [17]. To the best of our knowledge, no data are available in the scientific literature considering the effects of these two different feeding managements on the behavioural activities of horses raised for meat production. The goal of the present study was to fill this gap in the literature. Specifically, the study aimed to evaluate whether feeding farmed horses a fibre-based diet (thus increasing the time dedicated to feeding) could constitute an effective and feasible solution to apply at the farming level to bring about improvements in horse welfare.

## 2. Materials and Methods

### 2.1. Animals and Animal Husbandry

The present study forms part of a larger research project focused on assessing and improving the welfare of horses reared for meat purposes through changes to their feeding management. It was carried out on the biggest horse breeding farm authorised for meat production in northern Italy. Details about the housing and management conditions on this farm have been described elsewhere [5]. In brief, the farm adopts an intensive farming system characterised by group pens and an intensive feeding programme based on a high-starch diet. The twenty-four pens are located inside a barn open on two sides and arranged in two rows of twelve, separated by the central feeding lane. The pens are enclosed by horizontal metal rail bars which also delimit the pens at the feeding lane. The floor has a concrete base covered with barley straw bedding, of which one fresh flake (around 15 kg) per pen was added over the permanent bedding once a day. Horses were not tied up and they did not have any access to a paddock area. Two adjacent group pens were used for the present study. Nineteen Bardigiano horses (12 females and 7 stallions) aged 14.3 ± 0.7 months (mean ± SD) were involved. The Bardigiano is an Italian native breed for appreciation of its rusticity [18]. The breed is a meso-brachymorphic type, the coat colour is bay, and its traditional uses were for agricultural work and meat production [19].

Upon their arrival at the farm, the horses were kept together in an outdoor paddock for 14 days and treated against internal parasites. They were then moved into the barn and randomly divided into two group pens (7 m × 9 m) that provided at least 6 m^2^/animal. The stocking density (m^2^/horse) was calculated according to the methods described in Raspa et al. [5,20] as the area of each pen divided by the mean height at withers of the horses within the pen. Once transferred to the indoor pens (day 0), the same horses remained together until the end of the fattening period (day 129), when they were then slaughtered. One group of horses was fed according to the breeder’s standard feeding management system, based on high amounts of a starch-rich concentrate plus hay (high concentrate group–HCG; 5 females and 4 stallions). The second group received a fibre-based diet based on fibre-rich pelleted feed plus hay (high fibre group–HFG; 7 females and 3 stallions). The HFG diet was planned by the researchers according to the nutritional requirements of horses suggested by the French Institute National de la Research Agronomique (INRA) [21]. The same hay batch (chemical composition, as fed: crude protein 6.62%, ether extract 1.03%, crude fibre 30.04%, and ash 6.23%) was used for both groups. Hay consumption was estimated at 6 kg/animal/day for HCG, and 8 kg/animal/day for HFG [16]. Only horses in the HFG were fed hay using slow-feed HDP (high-density polyethylene) twine hay nets with mesh openings of 4 cm. Each horse received their individual ration of concentrates twice daily (7 am and 6 pm); horses in HCG received 8 kg/animal/day of the starch-rich concentrate pelleted feed (chemical composition, as fed: crude protein 14.21%, ether extract 3.69%, crude fibre 4.44%, ash 8.30%, and starch 49.50%), which provided 95.88 MJ/day net energy; whereas those in HFG received 3.5 kg/animal/day of the fibre-rich pelleted feed (chemical composition, as fed: crude protein 19.77%, ether extract 5.06%, crude fibre 11.53%, ash 10.78%, and starch 19.11%), providing 53.58 MJ/day net energy.

### 2.2. Behavioural Observations

A single 2D camera equipped with infrared light (D-Link DSH-C310 180°, Full HD) was installed in each pen. Behavioural observations were continuously recorded over a 96-h observation period (i.e., four consecutive days), corresponding to days 116, 117, 118, and 119 of the 129-day fattening period. The videos were evaluated by the same trained operator expert in equine behaviour using the ethogram published in Raspa et al. [20] (Table 1). The behavioural activities expressed by each horse were assessed by scan sampling the videos [22] at 10-min intervals throughout the entire 96-h observation period.

### 2.3. Data and Statistical Analysis

Statistical analyses were carried out using JMP v16.0 (SAS Institute Inc., Cary, NC, USA). To investigate the time–budget pattern, we assessed the daily scans for each behavioural activity and calculated their mean frequency (%/24 h) ± standard error of the mean (SEM). Frequency (%) ± SEM for the selected behavioural activities were compared between the two groups (HCG vs. HFG). All the behavioural data were checked for normality using the Shapiro–Wilk test and considered to adhere to a normal distribution for *p* > 0.05 [23]. Normally distributed data were reported as means ± SEM and analysed by Student *t*-tests. Those not abiding to a normal distribution were reported as medians (plus 25th–75th percentile) and analysed using the Mann–Whitney non-parametric test. The significance level was set at *p* > 0.05 [24].

Principal component analysis (PCA) (correlation matrix) was used to reduce the variables to factors [25]; data assumption for multivariate normality was checked using Keiser-Meyer-Olkin (KMO) and Barlett tests, which were performed to test the suitability of the data for structure detection. Only the principal components with eigenvalues higher than 2.5 were considered in the discussion [26].

A K-means cluster analysis was performed to visually show the spatial distribution of the HCG and FCG horses on PC1 and PC2 [27].

## 3. Results

The mean ± SD bodyweight (BW) of horses in HCG was 347.67 ± 6.71 kg; whereas the BW of horses in HFG was 344.40 ± 2.91 kg. The average daily bodyweight gain (ADG) was 1.01 ± 0.06 kg for HCG, and 0.96 ± 0.05 kg for HFG. In accordance with our previous results [16], no significant significance between groups was found in BW and ADG according to dietary treatment, sex, or their interaction.

A total of 144 behavioural observation scans of each horse were taken per day, providing a total of 10,368 scans sampled over the 96-h of video-recordings. A summary of the behavioural activities exhibited by the two horse groups (HCG vs. HFG) in terms of the percentage of time spent engaged in each behaviour is presented in Table 2. The percentage of time spent feeding was greater in HFG than HCG (40.21 ± 0.69% vs. 25.77 ± 0.38%, *p* < 0.01), and it constituted the daily behavioural activity most engaged in by horses belonging to HFG. On the contrary, the main behavioural activity performed by horses in HCG was standing, which occupied 30.29 ± 0.60% of their time compared with 24.82 ± 0.57% in HFG (*p* < 0.01). Locomotion was also observed more frequently in horses belonging to HCG than HFG (13.63 ± 0.61% vs. 7.44 ± 0.77%, *p* < 0.01). Similarly, playing behaviour was more frequent in the horses belonging to HCG than HFG (3.06 ± 0.10% vs. 1.92 ± 0.07%, *p* < 0.01), as was stereotypic behaviour, which considered both oral and locomotor stereotypies (0.38 ± 0.04% vs. 0.07 ± 0.01%, *p* < 0.01), and biting (0.08 ± 0.02% vs. 0.02 ± 0.01%, *p* = 0.02). On the other hand, although snaking was occasionally observed in horses belonging to HFG (0.08 [0.00–0.23]% of time, *p* < 0.01), this behavioural activity was never recorded in HCG.

**Figure 1:**
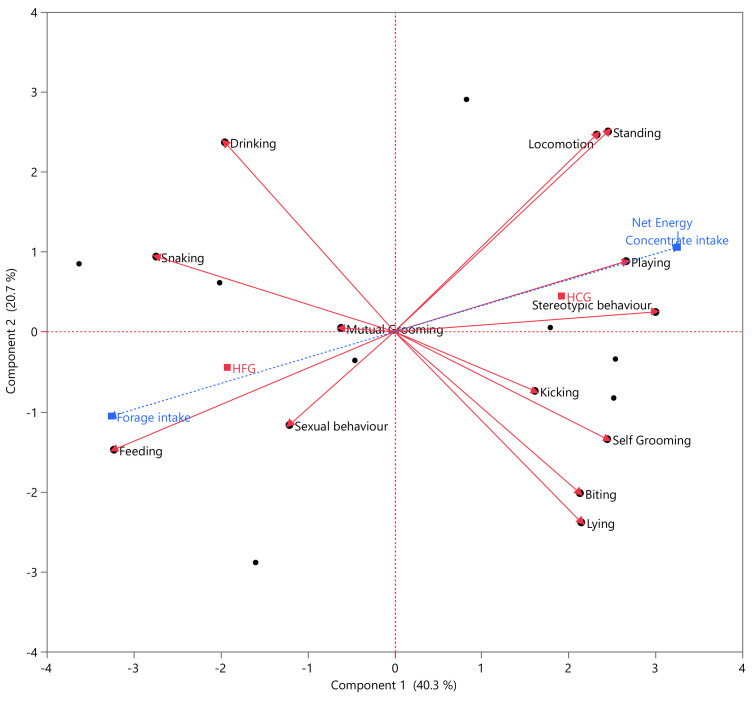
Principal component analysis biplot (PC1 and PC2) performed on selected behavioural activities by horses belonging to HCG and HFG.

Principal component analysis (PCA) was performed to explain the variability in the behavioural activities and to correlate each behavioural activity according to the dietary characteristics, i.e., concentrate intake, energy intake, and forage intake. The suitability of the data for PCA was evaluated (KMO = 0.80; Barlett’s test, *p* < 0.01). Figure 1 shows that PCA separated the dietary characteristics on the first principal component (PC1): component 1 explains 40.29% of the variance of the data, and component 2 (PC2) another 20.67%, for a total of 60.96% of variability. Table 3 shows the loadings of the variables of the first and second principal components, and how each variable contributes to each component. In particular, PCA showed that the higher intake of concentrates was positively correlated with a higher energy intake and with a lower forage intake. Moreover, the high intake of concentrates was positively correlated with more time spent self-grooming, lying, playing, locomotion, standing, kicking, biting, and stereotypic behaviour. Conversely, the diet based on a high forage intake was positively correlated with more time spent feeding, drinking, snaking, and sexual behaviour.

**Figure 2:**
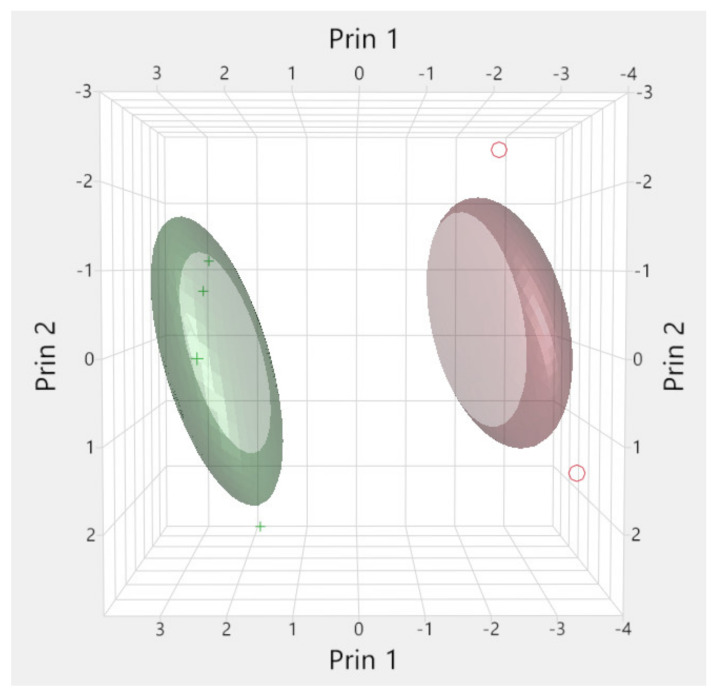
Cluster analysis 3D plot (PC1 and PC2) performed on the selected behavioural activities by horses belonging to HCG (red) and HFG (green).

K-means cluster analysis was performed to visually show the spatial distribution of the HCG and HFG horses on PC1 and PC2. Figure 2 shows that clustering the subjects according to their behavioural activities and dietary parameters distinguish the two groups (HCG and HFG) very effectively, confirming PCA results.

## 4. Discussion

The data presented here show that the feeding management system adopted can have important consequences on the expression of behavioural activities and thus welfare in horses reared for meat production. Feeding a fibre-based diet resulted in a greater proportion of the day horses spent feeding, and thus represents an effective and feasible solution to improve the welfare of horses reared in intensive systems for meat production. In fact, the main behavioural activity engaged in by horses belonging to HFG was feeding, which was expressed for 40.21 ± 0.69% of the time–in line with values reported for young (i.e., 2–3-year-old) wild-living horses, such as the Przewalski horses studied by Boyd et al. [28] shown to spend 46.4% of their day feeding. On the contrary, in the horses belonging to HCG feeding behaviour was only observed for 25.77 ± 0.38% of the time. The higher amounts of the time spent feeding in HFG was due to the provision of larger quantities of hay in slow-feeding hay nets. This point is particularly important from the welfare point of view since, according to the latest recommendations on forage feeding for horses, forage should be offered ad libitum or supplied throughout the day in order to avoid more than 4–5 h without foraging opportunity [29]. In particular, the lower limit of daily forage intake should be 15 g DM/kg BW in addition to complementary feeding [30]. It should also be considered that the foraging behaviour of horses is tightly linked to walking long distances under natural living conditions. Instead, the environmental constraints imposed by the breeding farm prevent the animals from expressing feeding behaviour while moving [31,32]. Importantly, one study found that keeping meat horses outdoors under conditions that permitted more walking behaviour resulted in better meat quality traits and more muscle gain [33]. Improved meat quality traits—e.g., a higher protein content of the muscle and a higher concentration of polyunsaturated fatty acids (PUFAs)—were also found in our horses belonging to HFG [16]. Therefore, although the lack of opportunity to move freely constitutes an important welfare concern in horses, increasing their foraging opportunity could provide at least one solution to apply at the farming level to improve both horse welfare and meat quality.

Compared with the horses belonging to HCG, those belonging to HFG spent less time engaged in standing behaviour (24.82 ± 0.57% vs. 30.29 ± 0.60%, respectively) and locomotion (13.63 ± 0.61% vs. 7.44 ± 0.77%, respectively). This finding is in agreement with the findings reported by Benhajali et al. [34], who showed that greater opportunity for foraging behaviour correlated with a reduction in standing in group-housed mares. The same study also proposed that less time spent in alert standing and in locomotion may correlate with lower stress levels in horses [34]. Therefore, the higher expression of both standing and locomotion in the HCG may be a sign of a lower welfare status.

A common perception is that excessive energy intake from concentrate feeds causes “fizzy” or unwanted excitable behaviours in horses, which would correlate with higher levels of locomotion and constitute a sign of agitation [35]. These so-called reactivity behaviours may be brought on by a starch-rich diet and the ensuing high glycaemic response they induce [8,36]. Accordingly, playing behaviour was also found to be expressed more frequently in HCG than in HFG (3.06 ± 0.10% vs. 1.92 ± 0.07%, respectively), although playing behaviour could also be related to immediate short-term positive emotions [9]. Interestingly, the horses in HCG also engaged in aggressive behaviour such as biting more frequently than those in HFG (0.08 ± 0.02% vs. 0.02 ± 0.01%, respectively). Biting is commonly associated with aggressive behaviour in situations involving competition between individuals, such as during foraging [37]. Thus, the higher incidence of biting in the HCG may be related to the reduced availability of hay. Moreover, the lack of foraging opportunity for horses belonging to HCG could explain why snaking behaviour was observed in HFG only, and absent in HCG. It may be that the snaking behaviour observed in horses belonging to HFG was related to the defence of their hay supply, which was always available.

The feeding management of horses Is strictly linked to equine gastrointestinal health, influencing both health and productivity [38]. It is well known that a diet based on high amounts of concentrates causes important changes in the gastrointestinal environment of the horse, and represents a risk factor for the onset of gastric ulcerations [24] and colic [7]. Among the induced changes, the increased production of total volatile fatty acids (VFAs), and specifically that of valeric acid caused by a high starch diet, has been shown to reduce mucosal integrity, leading to inflammation processes in the stomach [39,40], small intestine, and hindgut [7,41]. Moreover, the intensive management condition—i.e., the high stocking density (m^2^/horse), the feeding management based on a starch-rich diet, and the lack of opportunity for free movement—causes stress, a known risk factor for gastric ulceration as well as for the onset of stereotypic behaviours in horses [42]. Indeed, compared with the horses in HFG, those from HCG were found to possess more severe gastric ulcerations localized in the glandular region of the stomach (unpublished data), which may also have contributed to the higher incidence of stereotypic behaviour in HCG compared with HFG (0.38 ± 0.04% vs. 0.07 ± 0.01%, respectively).

Interestingly, other behavioural activities included in the ethogram—e.g., mutual grooming, self-grooming, kicking—were only detected at very low frequencies or were absent altogether. The sampling method used may have influenced the results, since scan sampling can miss some details—the duration of behaviours, and the behaviours exhibited in the intervals not analyzed—above all the behavioural activities occurring at low frequencies [5,43]. However, the plus side of this methodology is the possibility to analyse all the horses over short time periods, i.e., the 10 min blocks [43]. Finally, PCA was used to indicate the components able to explain the variability in behavioural activities according to dietary characteristics. The cluster analysis confirmed the results obtained from PCA. In particular, the horses in our HCG could be described as “excitable” [25] since consumption of the starch-rich diet was positively correlated with a higher expression of standing (i.e., alert standing), locomotion, playing, biting, and stereotypic behaviour. Conversely, we could describe the horses in HFG as “quiet” since this fibre-based diet was correlated with a higher expression of feeding and, accordingly, with a lower expression of the excitable behaviours listed above for the HCG. Furthermore, providing high amounts of forage is an important form of environmental enrichment for horses, as stated by Jørgensen et al. [44]. In summary, the feeding management based on a high-fibre diet resulted in animals that were calmer and less excitable. This aspect is also relevant from an economic point of view since, as documented in our previous publication related to this study [16], no significant differences in BW and ADG were found in the two groups of horses according to diet, sex, or their interaction. Feeding horses a fibre-based diet allows horses to spend more time expressing feeding behaviour, an important factor in improving horse welfare. Moreover, increasing the opportunity to forage reduces the time and energy spent expressing excitable behaviours. Therefore, the economic repercussions of the feeding management strategy adopted should be taken into account, and at the same time, solutions to increase the opportunity for free movement in horses reared for meat purposes should be considered in order to increase their welfare further.

## 5. Conclusions

The present study showed that the feeding management adopted for horses reared for meat production affects the repertoire of behavioural activities expressed, the related time budget and, as a consequence, their welfare. Feeding horses reared for meat production a fibre-based diet improves the welfare of these animals by increasing the time they spend engaged in feeding behaviour and reducing the frequency of behavioural activities identified as “excitable”. A fibre-based diet is thus advantageous from both a welfare and an economic point of view since it allows horses to express a more natural time budget, involving more time engaged in feeding behaviour, and reduces energy loss through the expression of excitable behaviours.

## Figures and Tables

**Table 1:** Descriptions of the evaluated behavioural activities. Adapted from Raspa et al. [20].

Behavioural Activities	Descriptions
Self-grooming	The horse cleans its body by shaking its entire body or a part of it, nibbling or licking the coat hair, rolling on the ground, or rubbing parts of the body against objects or other parts of the body (e.g., rubbing the muzzle against the limbs)
Mutual grooming	Body cleaning is performed reciprocally, or by one horse towards a conspecific
Lying	The horse is lying on the ground in the sternal position or in lateral position
Playing	The horse plays alone or with other horses. It includes: play with structural parts of the pen, sexual play, locomotor play, and play fighting
Locomotion	The horse moves inside the pen by taking steps; the neck is in a horizontal position or lowered to the ground to sniff
Feeding	The horse eats hay, straw, or feedstuff in the trough or on the ground
Drinking	The horse drinks
Standing	The horse is in a quadrupedal station. The expression is relaxed (standing relaxed) or attentive (standing alert)
Snaking	The horse stretches its neck towards a conspecific with the ears turned backwards, threatening to bite
Kicking	The horse lifts one or both hind limbs off the ground and quickly stretches it/them towards a conspecific
Biting	The horse quickly opens and closes its mouth, and its teeth touch the body of a conspecific. Its ears are turned backwards
Sexual behaviour	The stallion sniffs or bites the female’s genitals. Or the stallion mounts the female: erection and penetration are present
Stereotypic behaviour	The horse expresses a stereotyped behaviour: both oral and locomotor stereotypes are considered

**Table 2:** Time budget (%/24 h) engaged in different behavioural activities (HCG vs. HFG). All data are expressed as means ± SEM, with the exception of snaking and kicking which are expressed as medians (plus 25th–75th percentiles).

Behavioural Activities	HCG	HFG	*p*-Value
Feeding	25.77 ± 0.38	40.21 ± 0.69	<0.01 *
Standing	30.29 ± 0.60	24.82 ± 0.57	<0.01 *
Lying	22.65 ± 1.19	20.82 ± 0.56	0.18
Locomotion	13.63 ± 0.61	7.44 ± 0.77	<0.01 *
Playing	3.06 ± 0.10	1.92 ± 0.07	<0.01 *
Drinking	1.68 ± 0.34	2.61 ± 0.32	0.06
Mutual grooming	1.52 ± 0.33	1.52 ± 0.35	0.99
Self-grooming	0.83 ± 0.20	0.33 ± 0.15	0.06
Stereotypic behaviour	0.38 ± 0.04	0.07 ± 0.01	<0.01 *
Sexual behaviour	0.09 ± 0.02	0.12 ± 0.04	0.47
Biting	0.08 ± 0.02	0.02 ± 0.01	0.02 *
Snaking	0.00 (0.00–0.00)	0.08 (0.00–0.23)	<0.01 *
Kicking	0.00 (0.00–0.02)	0.00 (0.00–0.00)	0.15

* Statistical significance: *p* < 0.05.

**Table 3:** Principal component analysis loadings of behavioural activities performed by horses belonging to HCG and HFG.

	PC1 (40.29%)	PC2 (20.67%)
Feeding	−39%	−25%
Standing	30%	42%
Lying	26%	−40%
Locomotion	28%	41%
Playing	32%	15%
Drinking	−23%	40%
Mutual Grooming	−7%	1%
Self-grooming	29%	−23%
Stereotypic behaviour	36%	4%
Sexual behaviour	−15%	−20%
Biting	26%	−34%
Snaking	−33%	16%
Kicking	19%	−12%

## Data Availability

The data presented in this study are available on request from the corresponding author.
